# Chloroplast markers for the Malvaceae and the plastome of Henderson’s checkermallow (*Sidalcea hendersonii* S.Wats.), a rare plant from the Pacific Northwest

**DOI:** 10.1186/s13104-023-06357-4

**Published:** 2023-05-23

**Authors:** Diana M. Percy, Sæmundur Sveinsson, Andrew Ponomarev, Ji Yong Yang, Quentin C.B. Cronk

**Affiliations:** 1 Department of Botany, University of British Columbia, V6T 1Z4 Vancouver, BC Canada; 2 Matís Ltd, Vínlandsleið 12 113, Reykjavík, Iceland; 3 Department of Biology, Langara College, V5Y 2Z6 Vancouver, BC Canada; 4 Beaty Biodiversity Museum, University of British Columbia, V6T 1Z4 Vancouver, BC Canada

**Keywords:** Chloroplast genome, Chloroplast microsatellite, Conservation, Hypervariable region, Gynodioecy, Plastomics, Phylogeography, *Sidalcea*, Malvaceae

## Abstract

**Objective:**

*Sidalcea* is a genus of flowering plants restricted to the west coast of North America, commonly known as checkermallows. Remarkably, of the ~ 30 recognized species, 16 are of conservation concern (vulnerable, imperilled or critically imperilled). To facilitate biological studies in this genus, and in the wider Malvaceae, we have sequenced the whole plastid genome of *Sidalcea hendersonii*. This will allow us both to check those regions already developed as general Malvaceae markers in a previous study, and to search for new regions.

**Results:**

By comparing the *Sidalcea* genome to that of *Althaea*, we have identified a hypervariable circa 1 kb region in the short single copy region. This region shows promise for examining phylogeographic pattern, hybridization and haplotype diversity. Remarkably, considering the conservation of plastome architecture between *Sidalcea* and *Althaea*, the former has a 237 bp deletion in the otherwise highly conserved inverted repeat region. Newly designed primers provide a PCR assay to determine presence of this indel across the Malvaceae. Screening of previously designed chloroplast microsatellite markers indicates two markers with variation within *S. hendersonii* that would be useful in future population conservation genetics.

**Supplementary Information:**

The online version contains supplementary material available at 10.1186/s13104-023-06357-4.

## Introduction

*Sidalcea* (Malvaceae: Malveae) is a genus of ~ 30 species restricted to western North America, commonly called checkermallows. The Malvaceae is a large family containing such diverse plants as cacao (*Theobroma cacao* L.), cotton (*Gossypium* spp.), linden trees (*Tilia* species) and kapok (*Ceiba pentandra* (L.) Gaertn.). *Sidalcea* is a member of the tribe Malveae which mainly comprises herbaceous plants and shrubs. A recent study of Malveae phylogeny [1] divides the Malveae into two major clades and places the *Sidalcea* alliance (*Sidalcea* along with the related *Callirhoe*) in clade B which also contains the *Malva* alliance (*Malva, Alcea, Lavatera, Althaea*, etc.). *Sidalcea* itself has been the subject of detailed phylogenetic and evolutionary study [2–5], with particular interest focused on the evolution and maintenance of the gynodioecious mating system found in at least nine *Sidalcea* species [6–8].

Many of the *Sidalcea* species are narrow endemics, or locally rare, and several are of conservation concern (Table 1). One of these is *Sidalcea hendersonii* S.Wats. (Henderson’s checkermallow). Although this is the most widespread of the checkermallows, occurring from Oregon to Alaska, it is locally restricted wherever it occurs [9]. It is commonest in Washington State, where it was first collected by Louis Henderson in 1887. It is extremely rare in Oregon, where it has declined sharply since 1950 and is now only known from the Siuslaw River estuary [9, 10]. In 2003, it was discovered in Alaska, where it is only known from a single very small population at one locality [9, 11, 12]. In British Columbia, it is only known from the extreme south east of the Province [6], the main centre being the estuary of the Fraser River, but it is also found in southern Vancouver Island and the Southern Gulf Islands. It is a wetland species of estuarine swamps or coastal marshes and is vulnerable to environmental change, such as coastal land use alterations, river channel embankment and the spread of alien plants such as *Lythrum salicaria* [13]. In consequence, in many regions it appears to be declining.

**Table 1:** Endangered *Sidalcea* species (status information is from NatureServe Explorer [23]

Scientific name [Common name]	Status	Distribution	Notes
*Sidalcea celata* (Jeps.) S.R.Hill [Dwarf checkermallow]	G2 – imperilled	California	Known only from three counties in northern California
*Sidalcea covillei* Greene [Owens Valley checkermallow]	G3 – vulnerable	California	Endemic to Owens Valley, California
*Sidalcea gigantea* G.Clifton, R.E.Buck & S.R.Hill	G3 – vulnerable	California	Recently described species
*Sidalcea hendersonii* S.Wats. [Henderson’s checkermallow]	G3 – vulnerable	Oregon, Washington, British Columbia, Alaska	Limited number of populations (c.60–70) although widely distributed
*Sidalcea hickmanii* Greene [Chaparral checkerbloom]	G3 – vulnerable	California	Restricted to the coast ranges of California
*Sidalcea hirsuta* A.Gray [Hairy checkermallow]	G3 – vulnerable	California	
*Sidalcea hirtipes* C.L.Hitchc. [Bristly-stem checkerbloom]	G2 – imperilled	Oregon, Washington	
*Sidalcea keckii* Wiggins [Keck’s checkermallow]	G2 – imperilled	California	Known only from two counties in coastal California
*Sidalcea malachroides* (Hook. & Arn.) Gray [Mapleleaf checkerbloom]	G3 – vulnerable	California, Oregon	Coast region
*Sidalcea multifida* Greene [Cutleaf checkermallow]	G3 – vulnerable	California, Nevada	
*Sidalcea nelsoniana* Piper [Nelson’s checkermallow]	G2 – imperilled	Oregon, Washington	Largely restricted to the Willamette Valley of Oregon
*Sidalcea pedata* Gray [Pedate checkermallow]	G1 – critically imperilled	California	Known only from c. 10 sites
*Sidalcea ranunculacea* Greene [Marsh checkermallow]	G3 - vulnerable	California	
*Sidalcea robusta* Heller ex Roush [Butte County checkermallow]	G2 – imperilled	California	Known only from approximately 20 sites
*Sidalcea setosa* C.L.Hitchc. [Edgewood Checker-mallow]	G3 - vulnerable	California, Oregon	
*Sidalcea stipularis* J.T.Howell & True [Scadden Flat checkerbloom]	G1 – critically imperilled	California	Known only from two sites

A previous study that used plastome sequencing of cacao (*Theobroma cacao* L.) to develop plastid microsatellite markers for cacao and other Malvaceae also tested these primers on four species of *Sidalcea* [14]. Four of these cacao markers amplified successfully in the *Sidalcea* species, and three were polymorphic. This suggests that chloroplast markers can be of wide cross-species utility in assaying haplotype variation in the Malvaceae as a whole and in *Sidalcea* species in particular. Such markers could be useful for phylogeographic studies, as well as for studying haplotype diversity in populations and in detecting interspecific hybridization. To extend the range of resources available for *Sidalcea*, a genus presenting numerous species of conservation concern, we have therefore sequenced the whole plastome of an exemplar, *Sidalcea hendersonii*, and screened individuals of this species with select plastid microsatellite markers.

## Methods

### DNA extraction and illumina sequencing, assembly and annotation

Leaf samples of two individuals (one female and one hermaphrodite) from a population of *Sidalcea hendersonii* near Vancouver, British Columbia were collected into silica gel. Additional sampling for microsatellite marker screening was done from leaf material taken from herbarium specimens in the UBC Beaty Museum (Table 2). All specimens have vouchers deposited at the UBC Beaty Museum herbarium (UBC) with collection and identifier information available through the UBC herbarium Beaty Museum database (see data availability statement below). Total DNA was extracted using a modified version of the CTAB method from [15]. Illumina sequencing followed methods described previously [16]. Briefly, NEXTflex™ (Bioo Scientific Crop, Austen, TX, USA) was used for library construction. Fragment selection (400 bp) used Agencourt AMPure Xp™ magnetic beads (Beckman Coulter Genomics, Danvers, MA, USA). The two libraries were each sequenced on a single lane using 0.2 flow cells on an Illumina HiSeq-2000 sequencer generating 100 bp paired-end reads, giving a raw yield of 4–5 Gb per library and a chloroplast coverage per library of > x1000. The reads were assembled into complete plastomes, separately for each library, using CLC Genomic Workbench v.7.0.2 (CLC Bio). The sequences were annotated using cpGAVAS [17] with *Althaea officinalis* (GenBank: NC034701) as reference. The two annotations were then cross-checked using CHLOROBOX tools GeSeq [18] and GB2sequin [19], and graphically visualized with OGDRAW [20].

**Table 2:** Screening results for four chloroplast microsatellite markers from [14]. Sex female [F] or hermaphrodite [H] is given for *Sidalcea hendersonii*. Additional information on samples can be obtained from the UBC herbarium Beaty Museum database (https://databases.beatymuseum.ubc.ca/). For each marker the nucleotide repeat sequence for Cacao is given in [ ]. Variation within *S. hendersonii* is highlighted in bold

*Sidalcea* species	Sample info [sex]	UBC Accession	CaCrSSR1 [Ca-CTTTA]	CaCrSSR2 [Ca-A]	CaCrSSR3 [Ca-TAAAAG]	CaCrSSR8 [Ca-T]
*hendersonii*	Ladner, BC [H]	V217284	349	**239**	209	**289**
*hendersonii*	Ladner, BC [F]	V217283	349	**239**	209	**289**
*hendersonii*	Port Alberni, BC [H]	V217280	349	**239**	209	**284**
*hendersonii*	Port alberni, BC [F]	V217281	349	**239**	209	**291**
*hendersonii*	Comox, BC [H[	V217288	349	**241**	209	**285**
*hendersonii*	Comox, BC [F]	V217289	349	**241**	209	**285**
*hendersonii*	Trial Island, BC [H]	V217291	349	**245**	209	**284**
*oregana*	Kamloops, BC	V214044	349	239	209	283
*oregana*	Kamloops, BC	V2299919	349	239	209	283
*oregana*	Crook County, OR	V195946	349	239	209	283
*campestris*	Tahoe City, UT	V162588	349	239	209	283
*glaucescens*	Lake Tahoe, CA	V54739	349	239	209	284
*nellsoniana*	Polk County, OR	V220901	354	239	209	290

### Sequence analysis and marker testing

To search for regions of the plastid genome that are variable in the Malveae, the sequence was compared to the *Althaea* plastome sequence using zPicture [21]. Based on observed length variation, we designed new primers using Primer-BLAST [22] to test for the presence of a 237 bp indel [DP-SID2-indelF: 5’ TCCCGATTCATGGATCTCTCG 3’, DP-SID2-indelR: 5’ TGCCTTTTCTATTGATTCCTACGG 3’] across three subfamilies of Malvaceae.

Putative polymorphic microsatellite regions were tested using four of the primer pairs given in [14] (Table 2). Forward primers were synthesized with the universal M13 sequence TGTAAAACGACGGCCAGT. PCR amplifications for the microsatellites were performed in a final volume of 15 µl, containing 25 ng of DNA, 1× reaction PCR buffer (10 mM Tris-HCl pH 8.3, 50 mM KCl), 2.5 mM of each dNTP, 1.5 mM MgCl2, 1U of Taq DNA polymerase (Fermentas Canada, Burlington, Ontario, Canada), 0.1 µM forward primer, 0.5 µM reverse primer, and 0.5 µM fluorescently labeled M13 primer. Products were visualized using an ABI 3730 automated DNA Sequencer (Applied Biosystems). Primers were tested on seven individuals of *Sidalcea hendersonii* from four populations, and three individuals of *S. oregana* (Nutt. ex Torr. & A.Gray) A.Gray from two populations as well as on three other species (*S. campestris* Greene, *S. glaucescens* Greene, and *S. nelsoniana* Piper) (Table 2).

## Results

### Plastome features and comparison with Althaea

On assembly, the chloroplast genome was retrieved as a single contig. The two assemblies (female and hermaphrodite) were identical. Although no haplotype polymorphism was discovered, the two independently sequenced and assembled samples being identical gives us strong confidence in the assembly. The female *Sidalcea hendersonii* plastome sequence has been deposited in GenBank (OP780018).

**Fig. 1:**
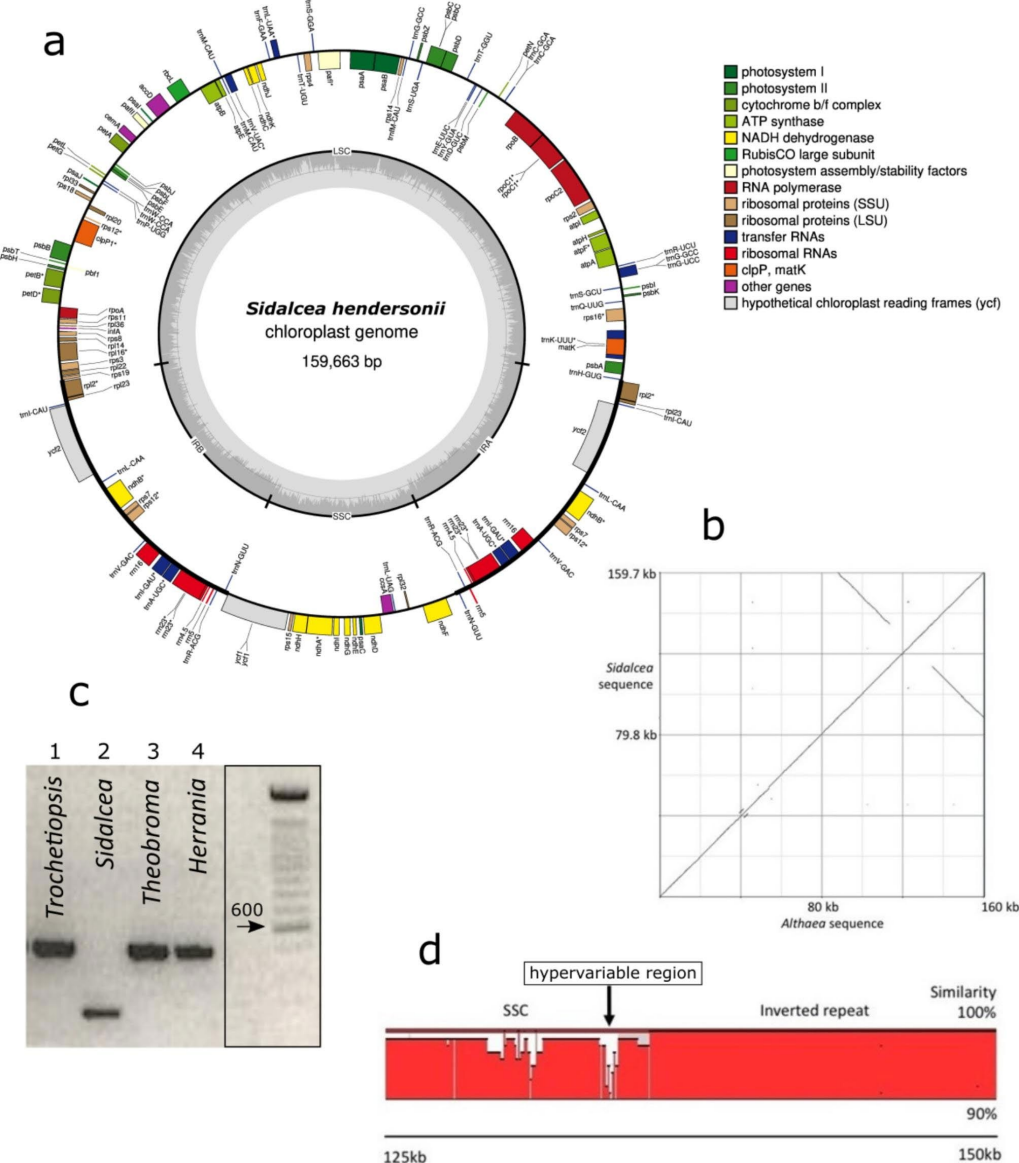
(a) Map of the *Sidalcea* plastome; (b) Dotplot showing the concordance between the *Althaea* and *Sidalcea* plastome structure; (c) Agarose gel of PCR bands using newly designed primers indicating *Sidalcea* as the only member across three Malvaceae subfamilies tested to have the 237 bp deletion (ladder moved up one lane, complete gel shown in Appendix 3); (d) Hypervariable region (arrowed, circa 1 kb) at the margin of the inverted repeat (IR) in the short single copy (SSC) region. The sequence similarity between *Sidalcea* and *Althaea* varies between 90–100% in 50 bp windows

The *Sidalcea hendersonii* plastome is 159,663 bp long (Fig. 1a), comparable to the 159,987 bp *Althaea officinalis* plastid genome. The structure and gene content are identical and there are no major rearrangements (Fig. 1b). This is despite the two species being in different clades of the tribe Malveae of the Malvaceae: the *Sidalcea* alliance vs. the *Malva* alliance. This suggests that members of the tribe Malveae have conserved plastid organization. Small indels are found, scattered through the genome, accounting for the 324 bp difference in overall length between the two species. The largest indel is a 237 bp deletion (Appendix 1) in the usually conserved inverted repeat (IR) in *Sidalcea* (around position 155,430 within ycf2). This deletion appears to be unique to *Sidalcea* (Fig. 1c), although we have not tested for it within the *Sidalcea* alliance clade. From sequences on GenBank, it is clear that other members of the Malvaceae subfamily Malvoideae (Hibisceae: *Hibiscus*, *Abelmoschus*, Malveae: *Althaea*) do not have this sequence deletion, neither do members of other subfamilies (Bombacoideae: *Bombax*; Tilioideae: *Tilia*; Byttnerioideae: *Theobroma*; Helicteroideae: *Durio*; Sterculioideae: *Firmiana*). Neither does this deletion appear to be present generally in rosid eudicots. The distribution of the deletion in the *Sidalcea* alliance remains to be more widely tested. We were able, with the newly designed primers [DP-SID2-indelF, DP-SID2-indelR], to confirm presence of the deletion in all four populations of *S. hendersonii* sampled (Table 2), as well as absence of the deletion in subfamily *Dombeyoideae: Trochetiopsis erythroxylon* (G.Forst.) Marais, (St Helena redwood), and subfamily Byttnerioideae: *Herrania balaensis* P. Preuss and *Theobroma cacao* (Fig. 1c). In addition, a few hotspots of variation were observed, with an especially hypervariable region at around 134,000 bp (c. 133,400–134,400 in *Sidalcea*; c. 133,600–134,600 in *Althaea*) in the short single copy (SSC) region (Fig. 1d). The alignment of this region is shown in Appendix 2. We consider this hypervariable region as a promising region in which to look for infraspecific and interspecific haplotype variation. Two of the four plastid microsatellites (CaCrSSR2 and CaCrSSR8) tested showed intraspecific variation within *Sidalcea hendersonii* (Table 2).

## Discussion

In making the plastome of *Sidalcea hendersonii* available we hope to stimulate further interest in this fascinating genus, containing as it does many species of critical conservation concern [23]. As has previously been pointed out [24], the lack of variation in many traditionally sequenced chloroplast regions has held back phylogeography in plants relative to animals. This has been somewhat alleviated by the ability to sequence multiple plastid genomes within a species [25, 26], but this is expensive. An alternative route, and the one used here, is to sequence one or a few plastomes per species and identify evolutionary hotspots, regions of elevated SNP variation of active homopolymer repeat stretches (“chloroplast microsatellites” [27]). This sort of variation is then amenable to standard, and cheaper laboratory methods. Such tools can produce conservation-relevant insights. For instance, the recent discovery of *S. hendersonii* disjunct in Alaska (noted above) raises the question of whether this is a result of long distance dispersal from southern populations or the survival of a population that may represent a divergent infraspecific evolutionary lineage and therefore merit particular conservation attention. The identification of haplotype variation as detailed here (e.g., the two polymorphic chloroplast microsatellites), could potentially help in answering this question, and others like it.

## Limitations

Current rarity and threatened status of *Sidalcea hendersonii* and other *Sidalcea* species presents difficulties in obtaining sufficient sampling for population analysis; additionally obtaining reasonable quality DNAs from historical samples in herbaria is also challenging.

## Electronic supplementary material

Below is the link to the electronic supplementary material.

Supplementary Material 1

## Data Availability

The annotated *Sidalcea hendersonii* plastid genome is publicly available from GenBank (OP780018). Additional sample information for herbarium specimens sampled for the chloroplast microsatellites is available from the UBC herbarium Beaty Museum database (https://databases.beatymuseum.ubc.ca/). All other data generated or analysed during this study are included in this published article [and its supplementary information files].
