# Supplementary material for: Chloroplast markers for the Malvaceae and the plastome of Henderson’s checkermallow (Sidalcea hendersonii S.Wats.), a rare plant from the Pacific Northwest
Source: BMC Res Notes. 2023 May 23;16:87. doi: 10.1186/s13104-023-06357-4 (PMC10207809; doi:10.1186/s13104-023-06357-4)

Chloroplast markers for the Malvaceae and the plastome of Henderson's checkermallow (*Sidalcea hendersonii* S.Wats.), a rare plant from the Pacific Northwest

Diana M. Percy (1)*, Sæmundur Sveinsson (2), Andrew Ponomarev (3), Ji Yong Yang (3), Quentin C.B. Cronk (1,4)

* Corresponding author. Email: diana.percy@ubc.ca

(1) Department of Botany, University of British Columbia, Vancouver BC, V6T 1Z4, Canada

(2) Matís Ltd, Vínlandsleið 12 113, Reykjavík, Iceland

(3) Department of Biology, Langara College, Vancouver BC, V5Y 2Z6, Canada

(4) Beaty Biodiversity Museum, University of British Columbia, Vancouver BC, V6T 1Z4, Canada

Supplementary material.

Appendix 1. Sequences from the inverted repeat showing the 237 bp indel (see text and Fig. 1).

```
Sidalcea_IR(part)      TATGCATTAGGATCCCGATTCATGGATCTCTCGGTCCGAGAAATCAAAATAAGAGGATCG
Althaea_IR(part)       TATGCATTAGGATCCCGATTCATGGATCTCTCGGTCCGAGAAATCAAAATAAGAGGATCG
                       ************************************************************

Sidalcea_IR(part)      AACCATTTCTTCTGACTCTTTTTCAAATTCGATAAATGTTGGTTGATCGTATATTTCATT
Althaea_IR(part)       AACCATTTCTTCTGACTCTTTTTCAAATTCGATAAATGTTGGTTGATCGTATATTTCATT
                       ************************************************************

Sidalcea_IR(part)      ATAGTTCTATGATTCAGAGTATCATTTCCTATTTGATCCCTTTGAATTCCATATTCGAAG
Althaea_IR(part)       ATAGTTCTATGATTCAGAGTATCATTTCCTATTTGATCCCTTTGAATTCCATATTCGAAG
                       ************************************************************

Sidalcea_IR(part)      TTGCGATCGGATCGATTCATTAAAAAGAATCGATTCA-----------------------
Althaea_IR(part)       TTGCGATCGGATCGATTCATTAAAAAGAATCGATTCAATACATTTCTTATGTACCCATAG
                       *************************************

Sidalcea_IR(part)      ------------------------------------------------------------
Althaea_IR(part)       GTGCTATATTGGATTTGAATCAGATTTCGGATCAATCTATCTTGATTGACTGCCTCCATT


Sidalcea_IR(part)      ------------------------------------------------------------
Althaea_IR(part)       ATGTTGTTGCTAGCAAATACCACTATTTTTGGTTTTGGATCTTCCAAATCATTCCCGCAG


Sidalcea_IR(part)      ------------------------------------------------------------
Althaea_IR(part)       GAGATCCGGACCCATTTTTTTCTGATCCTTCGATAAAAAGATTCATTTTCTTCATAAAAA


Sidalcea_IR(part)      ----------------------------------ATTCATCCCTGGAGTTGAATACCTCA
Althaea_IR(part)       ATAGGAGGTAGAACCGATAAAGATTTCTTTTTCGATTCATCCCTGGAGTTGAATACCTCA
                                                         **************************

Sidalcea_IR(part)      TTCAAGAATTGTTTTTGATCCAATCCGTAGGAATCAATAGAAAAGGCAAATCCCTTATGA
Althaea_IR(part)       TTCAAGAATTGTTTTTGATCCAATCCGTAGGAATCAATAGAAAAGGCAAATCCCTTATGA
                       ************************************************************

Sidalcea_IR(part)      TACACCAGATCCGGCTCGGTTATTGATAGAGTGAATAGATCTGCCATTTCTTGAAATCTC
Althaea_IR(part)       TACACCAGATCCGGCTCGGTTATTGATAGAGTGAATAGATCTGCCATTTCTTGAAATCTC
                       ************************************************************

Sidalcea_IR(part)      TCTTCTGATTCAAAATCGTGGTGTAACGTGTATCCCCCCCTGTTCTGGTCATGGAATAGA
Althaea_IR(part)       TCTTCTGATTCAAAATCGTGGTGTAACGTGTATCCCCCCCTGTTCTGGTCATGGAATAGA
                       ************************************************************

Sidalcea_IR(part)      TGAAATAAATCAAAAAATGGATTTTTGTTCAAGAATGAAATCTTATTGGAACTGCCCAGT
Althaea_IR(part)       TGAAATAAATCAAAAAATGGATTTTTGTTCAAGAATGAAATCTTATTGGAACTGCCCAGT
                       ************************************************************
```

Appendix 2. Hypervariable region in the small single copy region (see text and Fig. 1)

```
Althaea_SSC(part)         ATATTATCAAAATTATTAACTCCATCTATAAATCTTTTACATCAAAATTCAAATGATTTT
Sidalcea_SSC(part)        ATATTATCAAAATTATTAACTCCATCTATAAATCTTTTACATCAAAATTCAAATGATTTT
                          ************************************************************

Althaea_SSC(part)         GAGGATTCATATCAATTTTTAACAAATGCAACTTTTTCGGTGAGTATAGCTTGTTTCGGA
Sidalcea_SSC(part)        GAGGATTCATATCAATTTTTAACAAATGCAACTTTTTCGGTGAGTATAGCTTGTTTCGGA
                          ************************************************************

Althaea_SSC(part)         ATATTTACAGCATTCCTTTTATATAAGCCTTTTTATTCATCTTTACAAAATTTGAACTTA
Sidalcea_SSC(part)        ATATTTACAGCATTCCTTTTATATAAGCCTTTTTATTCATCTTTACAAAATTTGAACTTA
                          ************************************************************

Althaea_SSC(part)         CTAAATTCGTTTGCGAAAAGGGGTCCTAAAAGGATTTTATTGGATAAAATAATATATTTG
Sidalcea_SSC(part)        CTAAATTCGTTTGCGAAAAGGGGCCCTAAAAGGATTTTATTGGATAAAATGATATATTTG
                          *********************** ************************** *********

Althaea_SSC(part)         ATATATGATTGGTCATATAATCGTGGTTACATAGATACGTTTTATTCAATAGCCTTAACA
Sidalcea_SSC(part)        ATATATGATTGGTCATATAATCGTGGTTACATAGATAGGTTTTATTCAATAGCCTTAACA
                          ************************************* **********************

Althaea_SSC(part)         AAAGGTATAAGGGGATTGGCCGAACTAACTCATTTTTTTGATAGGCGAGTAATCGATGGA
Sidalcea_SSC(part)        AAAGGTATAAGAGGATTGGCCGAACTAACTCATTTTTTTGATAGGCGAGTAATCGATGGA
                          *********** ************************************************

Althaea_SSC(part)         ATTACAAATGGAGTAGGCATTACAAGTTTTTTTGTAGGAGAAAGCATAAAATATGTAGGA
Sidalcea_SSC(part)        ATTACAAATGGAGTAGGCATTACAAGTTTTTTTGTAGGAGAAAGCATAAAATATGTAGGA
                          ************************************************************

Althaea_SSC(part)         GGAAGTCGCATCTCGTTTTATCTATTATTGTATTTATTTTATGTATTAATTTTTTTAGTA
Sidalcea_SSC(part)        GGAAGTCGCATCTCGTTTTATCTATTATTGTATTTATTTTATGTATTCATTTTTTTAGTA
                          *********************************************** ************

Althaea_SSC(part)         ATTTACTACTTTATTCTATTTTAATTT--TTTTTCTACAGCTCCTTGCTCTGGATGTTGT
Sidalcea_SSC(part)        ATTTACTACTTTATTATATTTTAATTTTTTTTTTCTACAGCTCCTTGCTCTGGATGTTGT
                          *************** ***********  *******************************

Althaea_SSC(part)         TCCCTCGGTGTGAAAGCAGTTGGTTCCGTAGTTTTAGAATTCCGCTGATCCCAAGTACTC
Sidalcea_SSC(part)        TCCCTCGGTGTGAAAGCAGTTGGTTCCGTAGTTTTCGAATTCCGCTGATCCCAAGTACTC
                          *********************************** ************************

Althaea_SSC(part)         CATCTACATCATTGCATAGAAGATCAAATA-GAAAATAAAGAGGAAAGGGCATAACCATA
Sidalcea_SSC(part)        CATCTACATCATTGCATAGAAGATCAATATAGAAAATAAAGAGGAAAAGGCATAACCATA
                          ***************************    **************** ************
```

```
Althaea_SSC(part)      AGAATTTTGAAAAATGCCTCACTTTATCCAATTGAGGCATTTTTCAAAACGATTCCCTAC
Sidalcea_SSC(part)     AGAATTTTGAAAAATGCCTCAAAA------GGTGAGGCATTTTTCAAAACGATTCCCTAC
                       *********************           ****************************

Althaea_SSC(part)      AGACAGGATG-------TACAAAAAAATTCGGAATGAGCGTACCGACGGACCTAGAGAGA
Sidalcea_SSC(part)     AGACAGGATCAGGATGGACAAAAAAAATTCGGAATAAGCGTACCGACGGACCTAGAGAGA
                       *********           *************** ************************

Althaea_SSC(part)      GGAACCCCCTCTCTCACCCGGCCCCCCTACCTAAACCGATCCTCATTATCGGTCCCTGGG
Sidalcea_SSC(part)     GGAACCCCACCCC-CTCTCTCTCACCCGACCGCCCTAGATCATCATTATCGGTCCCTGGG
                       ********  * * * * *   * *** ***      **** ******************

Althaea_SSC(part)      TCCCATTACATTACTTTACCGGCTTTACTGGGCTTTCCTAAATTTCAGCCTTAACTTAAG
Sidalcea_SSC(part)     TCCCATTACATGACTTTACCGGCTTTACTGGGCTTTCCTAAATTTCAGCCTTAACTTAAG
                       *********** ************************************************

Althaea_SSC(part)      GGGA-ACGTCGTTTTTTAAGAGCTCAAAGGCCTGGGAATTTTTGCCGTTCTTCTCGAGAT
Sidalcea_SSC(part)     GGGGAACGCCGTTTTTTAACAGCTCAAAGGCCTGGGAATTTTTTCTGTTCTTCTAGAGAT
                       ***  *** ********** *********************** * ******** *****

Althaea_SSC(part)      CTTTATACTTTATAAATATAACCTAGCTTTGTTAACGAGGCCTCTGTCAACTCCGTCTTG
Sidalcea_SSC(part)     CTT-------TATAAATATAACCTAGCTTTGTTAACGAGGCCTCCGTCAACTCCGTCTTG
                       ***       ********************************** ***************

Althaea_SSC(part)      CCCATATTATTCATGGCGCGAGCTGCCTGCCCCCTCCAATCTCCCAGTGAGTCAAGGGGT
Sidalcea_SSC(part)     CGCATATTATTCATGACGTGAGCTGCCTGCCCCTCCAAT-CTCCCAATGGGTCAAGGGGC
                       * ************* ** **************  * *  ****** ** *********

Althaea_SSC(part)      TCCCTAAGGTTTATAATCTTTTCCTTTTGTCGCAAAGCCTTTCCGCCTTTTTCTCAGTCT
Sidalcea_SSC(part)     -TCCCGAGGTTTATAATCTTTTCTTTTTGTCGCAAAGCCTTTTGGCCTTTTT--TTTTAT
                         **  ***************** ******************  ********     * *

Althaea_SSC(part)      CAGCCACGACTGTCTCGTCAGTCGGGTATCTGTACCCAAAAAACGGATTCATGATCAGCT
Sidalcea_SSC(part)     CAGCCACGACTGTCTCGTCAGTCGGGTATCTGTACCAAAAAAACGGATTCACGATCAGCT
                       ************************************ ************** ********

Althaea_SSC(part)      AGACCGAATCCCCCCCCCTATCCTCTCATCCAGTTGATACGGAGAGCGTCTCGAATCCTC
Sidalcea_SSC(part)     AGACCGAAGCCC--CTCCTATCCTCTTATCCAGTTGATACGGAGAGCGTCTCGAATCCTC
                       ******** ***  * ********** *********************************

Althaea_SSC(part)      TTGTGGGGGAGGATTTGCTGGACCAGCTTCGTCCCCGGGGGGAGCACCTTGATTAGAAGT
Sidalcea_SSC(part)     TTGTGGGGGAGGATTTGATGGACCAGCTTCGTCCCCGGGGGGAGCAC---------AAGT
                       ***************** *****************************         ****

Althaea_SSC(part)      TGTGGTGTTGTCGTCCCACAAAAAGTTACGCGTAATCAGCCTATGTCCAGATTCTGGCTG
Sidalcea_SSC(part)     TGTGGTGTTGTCGTCCTACAAAAAGTTACGCGTAATCAGCCTACGTCCAGATTCTGGCTG
```

```
                                  **************** ************************** ****************

Althaea_SSC(part)        ATTAAACGACGTGTTCGTT----------------------------------------T
Sidalcea_SSC(part)       ATTAAACCATGTGTTCGTTTATATTTCGTTTATATTATATTTCGTTTATATTTATAATTT
                         ******* * *********                                        *

Althaea_SSC(part)        ATAGGTGCTACCTCCTTTTTTCTTTTCTATTTCTGGATTATTATATGATGATTTTTGAAC
Sidalcea_SSC(part)       ATAGGTGATACCTCCTTTTTTCTTTTCTATTTCTGGATTATTATATGATGATTTTTGAAC
                         ******* ****************************************************

Althaea_SSC(part)        TTTCCATATACATATATAGAAAAAGATAGACTAGAACCGACATCTCTTATGTCATGTCAA
Sidalcea_SSC(part)       TTTCCATATACATATATAGAAAAAGATAGACTAGAACCGACATCTCTTATGTCATGTCAA
                         ************************************************************

Althaea_SSC(part)        TGACAATATAAAAATGGAATTGGGATCTGGATGGAATATAATGAAATAGAGCCACTTTGA
Sidalcea_SSC(part)       TGACAATATAAAAATGGAATTGGGATCTGGATGGAATATAATGAAATAGAGCCACTTTGA
                         ************************************************************

Althaea_SSC(part)        GGTTCCCTATGAAATGAGGCATGGAACGGAGCCACTACGAAGAAGTTCCGGGG
Sidalcea_SSC(part)       GGTTCCCTATGAAATGAGGCATGGAACGGAGCCACTACGAAGAAGTTCCGGGG
                         *****************************************************
```

Appendix 3. Complete agarose gel indicating part (boxed) shown in Fig. 1 to illustrates PCR test for 237 bp deletion (see text).

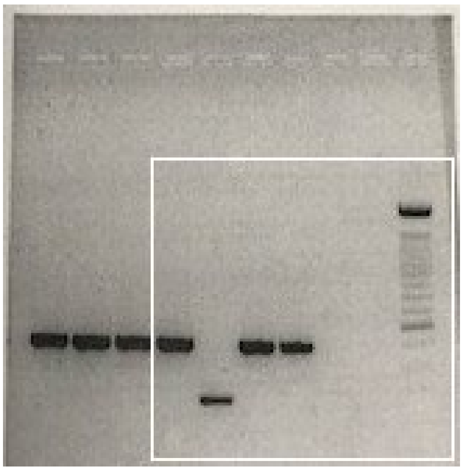

Supplement: Supplementary file 1 — Supplementary Material 1 [file 13104_2023_6357_MOESM1_ESM.pdf]
